# Association between lipid ratio and depression: a cross-sectional study

**DOI:** 10.1038/s41598-022-10350-5

**Published:** 2022-04-13

**Authors:** A Lum Han

**Affiliations:** Department of Family Medicine, Wonkwang University Hospital, Sinyong-dong 344-2, Iksan, Jeollabuk-do 54538 Korea

**Keywords:** Psychology, Health care, Medical research

## Abstract

Depression is associated with total cholesterol, low-density lipoprotein cholesterol, triglycerides, and high-density lipoprotein cholesterol levels in the blood. However, evidence is limited on the relationship between depression and lipid ratios. Therefore, this study aimed to investigate the correlation between depression and different lipid ratios. This study was conducted using data from the Korea National Health and Nutrition Examination Survey. A total of 11,648 adult men and women aged ≥ 19 years, without missing data, were included in this study. Depression was diagnosed using the Patient Health Questionnaire-9. The associations between depression and total cholesterol/high-density lipoprotein cholesterol, low-density cholesterol/high-density lipoprotein cholesterol, and triglyceride/high-density lipoprotein cholesterol ratio were analyzed. A complex sample logistic regression test was used for the analysis of the odds ratios of depression. Among men, the total cholesterol/high-density lipoprotein cholesterol and low-density cholesterol/high-density lipoprotein cholesterol ratios were not associated with depression. Additionally, an increase in triglyceride/high-density lipoprotein cholesterol ratio by 1 was associated with a 1.041-fold higher probability of depression in men. Among women, the three lipid ratios were not associated with depression. Triglyceride/high-density lipoprotein cholesterol ratio is associated with depression among men. Future studies should cross-validate, explore the biological mechanism, and identify the clinical implication of this correlation.

## Introduction

Depression is a common mental disorder characterized by sadness, loss of interest or pleasure, feelings of guilt or low self-esteem, trouble sleeping or eating, fatigue, and difficulty concentrating^1^. Depression is often chronic and impairs quality of life. It may be associated with other chronic diseases and can lead to an increased risk of death. According to the World Health Organization, the prevalence of depression is increasing worldwide and is predicted to become the most prevalent disease by 2030^1^.

The relationship between low cholesterol levels and depression has been investigated in several previous studies. It is believed that low cholesterol levels influence depression by altering the metabolism of serotonin^2,3^. Depression causes increased food intake, increase in body weight, and decreased frequency of exercise, and it also elevates blood cortisol level, which leads to impaired glucose tolerance, high blood pressure, fat accumulation, and eventually, metabolic syndrome^3^. In addition, depression can adversely affect lipid metabolism^3^. Therefore, some studies have shown a correlation between depression and elevated triglyceride (TG) and low high-density lipoprotein cholesterol (HDL-C) levels^4,5^.

Several studies have been conducted to analyze the relationship between depression and cholesterol levels. However, there are only a few studies on the relationship between lipid ratio and depression. Furthermore, previous studies have mainly focused on the relationship between lipid ratio, cardiovascular disease, and insulin resistance. In addition, depression is associated with obesity and metabolic syndrome^6,7^, and the association of depression with TG and HDL-C levels has been evaluated previously^5^. Based on these studies, the association between the lipid ratio and depression may be inferred, which can emphasize the role of lipid metabolism in depression. Therefore, the aim of this study was to analyze the correlation between depression and three lipid ratios, namely total cholesterol (TC)/HDL-C, LDL-C/HDL-C, and TG/HDL-C, in a large population.

## Methods

### Aim, design, and setting

This large-scale cross-sectional study was conducted to analyze the association between depression and lipid ratios. This study was conducte in accordance with the ethical standards of the Declaration of Helsinki and obtained approval from the Wonkwang University Hospital’s clinical trial screening committee (IRB, approval number 2021-09-001).

### Participants and enrollment

**Figure 1:**
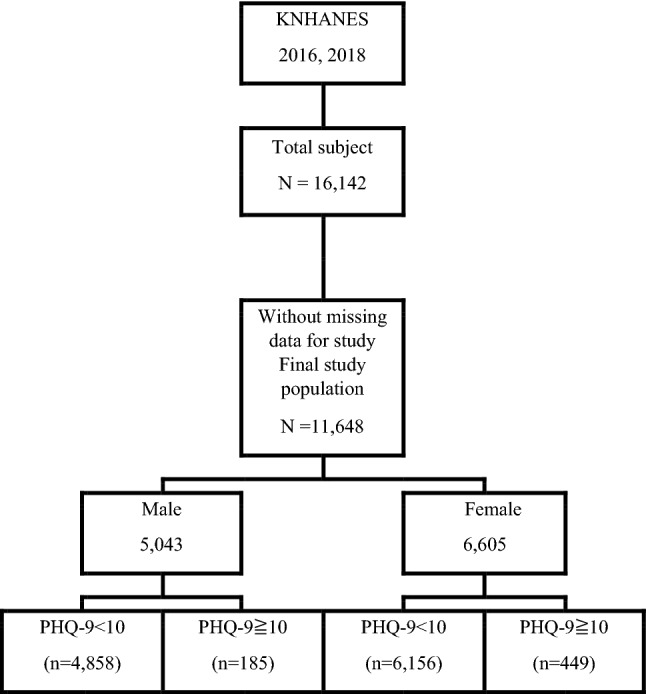
Flow diagram of the participants. *PHQ-9* Patient Health Questionnaire-9, *KNHANES* Korea National Health and Nutrition Examination Survey.

This study was conducted using data from the Korea National Health and Nutrition Examination Survey (KNHANES). We analyzed the data generated in the 2016 and 2018 surveys when the Patient Health Questionnaire (PHQ)-9, which is a diagnostic tool for depression, was used. Only male and female participants aged 19 years and older and without any missing data were included. Participants who completed all nine items of the PHQ-9 were analyzed (Fig. 1).

### Anthropometric and biochemical parameters

All tests were performed by trained examiners. Height (cm) and weight (kg) were measured using a Seca 225 machine (Seca, Hamburg, Germany) and a GL-6000-20 scale (G-tech, Seoul, Korea). Body mass index was calculated as weight (kg) divided by height squared (m^2^). Systolic blood pressure and diastolic blood pressure were measured twice or more at 10-min intervals using a mercury sphygmomanometer (Baumanometer; WA Baum Co., Copiague, NY) wrapped around the right upper arm and the average value was recorded. Blood samples were collected after an 8-h fasting period. Fasting blood glucose (FBG), hemoglobin A1c, TC, TG, HDL-C, LDL-C, and hemoglobin levels were measured using the Hitachi 7600 automatic analyzer (Hitachi, Tokyo, Japan).

### Sociodemographic variables

The smoking and drinking history, degree of physical activity, educational level, and household income of the participants were evaluated. Based on smoking and drinking history, participants were classified as current smokers or non-smokers and as current drinkers or non-drinkers. An international physical activity questionnaire was used to measure the degree of physical activity. Exercising five or more times a week for 30 min or performing vigorous physical activity three times or more for ≥ 20 min was considered regular exercise. Activities such as slow swimming, doubles tennis, volleyball, badminton, table tennis, and light lifting were defined as appropriate physical activity. Participants were divided into four groups according to their educational background: elementary school graduate, middle school graduate, high school graduate, and college graduate or higher. Average gross household income was divided into four levels: low (less than 1 million won per month), medium–low (1 to 2 million won per month), medium (2 to 3 million won per month), and high (3 million won or more).

The degree of percieved stress was assessed based on the following choices: (1) I feel stressed all the time, (2) I feel stressed a lot, (3) I feel stressed sometimes, and (4) I hardly feel stressed.

Responses 1 to 3 showed that the participants perceived stress, and response 4 indicated a lack of stress. In the tables, ‘Y’ represents prerceived stress and ‘N’ represents absence of stress.

### Screening for depression

Screening for depression in large populations requires the use of an economical, efficient, and time-consuming tool, such as the PHQ-9^7–9^. The PHQ-9 has the advantage of providing information on the severity of symptoms and includes a diagnostic algorithm that evaluates the presence or absence of major depressive disorder within a short period^10^. The time required for testing is relatively short, and the reliability and validity of the tool have been consistently demonstrated to be satisfactory in many studies^11,12^. Several studies have suggested that a score ≥ 10 in the PHQ-9 indicates suspected depression^7–9^. A Korean study that proved the reliability and validity of PHQ-9, set the cut-off value at ≥ 10^9^. Therefore, in this study, the relationship between depression and each lipid ratio was analyzed with PHQ-9 as a diagnostic tool for depression. The PHQ-9 is a nine-item questionnaire on the symptoms associated with depressive disorder. Each item was scored from 0 to 3 depending on the severity of the symptoms. Subsequently, total score was calculated and recorded. We classified a PHQ-9 score ≥ 10 as depression, and scores below it as absence of depression.

### Statistical analysis

Statistical analysis was performed using PASW (SPSS version 26.0, IBM SPSS Inc., Chicago, IL, USA). Statistical significance was set to *p* < 0.05. Frequency analysis was performed using a complex sample plan. After categorization according to sex, we analyzed the relationship between general characteristics, blood test results, and PHQ-9 scores using a complex sample Rao–Scott adjusted chi-square test and a complex sample generalized linear model (Tables 1, 2). Odds ratios (OR) and 95% confidence intervals were estimated by adjusting for statistically significant variables. Variables that could be affected were adjusted for age, sex, education level, household income, and drinking and smoking history. Thereafter, a complex sample logistic regression test was used for analysis (Table 3).

**Table 1:** Differences between men with and without depression.

Variable	Men (n = 5043)		
	PHQ-9 < 10 (n = 4858)	PHQ-9 ≧ 10 (n = 185)	*p*
Age	45.96 ± 0.31	45.92 ± 1.28	0.972
**Household income**			
Low	1147 (24.8)	96 (51)	< 0.0001
Middle-low	1219 (25.2)	42 (21)	
Middle-high	1253 (25.4)	19 (11.8)	
High	1239 (24.6)	28 (16.2)	
**Education level**			
Elementary	660 (94.1)	49 (5.9)	0.031
Middle	490 (97)	17 (3)	
High	1703 (97)	60 (3)	
College	2017 (97.1)	58 (2.9)	
**Alcohol consumption**			
N	1448 (97.1)	55 (2.9)	0.385
Y	3417 (96.6)	130 (3.4)	
**Stress perception**			
N	3792 (76)	60 (24.4)	< 0.0001
Y	1073 (24)	125 (75.6)	
**Smoking**			
N	3199 (63.2)	94 (45.3)	< 0.0001
Y	1665 (36.8)	91 (54.7)	
Height (cm)	171.55 ± 0.13	170.76 ± 0.49	0.118
Weight (kg)	72.5 ± 0.2	71.96 ± 1.15	0.647
BMI (kg/m^2^)	24.59 ± 0.06	24.63 ± 0.38	0.919
Fasting glucose (mg/dL)	102.49 ± 0.47	109.26 ± 3.36	0.046
SBP (mmHg)	120.15 ± 0.28	120.88 ± 1.22	0.554
DBP (mmHg)	78.56 ± 0.19	76.27 ± 1.00	0.023
HbA1c (%)	5.67 ± 0.02	5.77 ± 0.08	0.278
TC (mg/dL)	191.71 ± 0.67	191.95 ± 3.23	0.943
HDL-C (mg/dL)	47.36 ± 0.21	47.03 ± 1.16	0.781
TG (mg/dL)	163.89 ± 2.92	203.01 ± 15.24	0.009
LDL-C (mg/dL)	117.99 ± 1.22	114.36 ± 5.7	0.542
Hb (g/dL)	15.38 ± 0.02	15.28 ± 0.11	0.352
TC/HDL-C	4.25 ± 0.02	4.61 ± 0.25	0.150
LDL-C/HDL-C	2.94 ± 0.03	3.17 ± 0.23	0.342
TG/HDL-C	3.94 ± 0.09	6.10 ± 1.03	0.035

‘Y’ represents presence of perceived stress, and ‘N’ represents absence of perceived stress.

‘Y’ represents current smokers and ‘N’ represents non-smokers.

‘Y’ represents current drinkers and ‘N’ represents non-drinkers.

Values are presented as number (%) or the mean ± standard deviation.

*p* value was determined through the complex sample Rao–Scott adjusted chi-square test and complex sample generalized linear model T test.

*PHQ-9* Patient Health Questionnaire-9, *BMI* body-mass index, *SBP* systolic blood pressure, *DBP* diastolic blood pressure, *HbA1c* hemoglobin A1c, *TC* total cholesterol, *HDL-C* high density lipoprotein cholesterol, *TG* triglyceride, *LDL-C* low density lipoprotein cholesterol, *Hb* hemoglobin.

**Table 2:** Differences between women with and without depression.

Variable	Women (n = 6605)		
	PHQ-9 < 10 (n = 6156)	PHQ-9 ≧ 10 (n = 449)	*p*
Age	47.99 ± 0.35	48.79 ± 1.21	0.505
**Household income**			
Low	1436 (24.3)	178 (39.8)	< 0.0001
Middle-low	1551 (25.3)	125 (27.2)	
Middle-high	1551 (25)	99 (22.3)	
High	1618 (25.4)	47 (10.8)	
**Education level**			
Elementary	1463 (89.6)	175 (10.4)	< 0.0001
Middle	619 (91.4)	62 (8.6)	
High	1943 (94.2)	119 (5.8)	
College	2142 (95.2)	94 (4.8)	
**Alcohol consumption**			
N	3586 (93.2)	275 (6.8)	0.474
Y	2570 (93.7)	173 (6.3)	
**Stress perception**			
N	4635 (74.4)	101 (20.4)	< 0.0001
Y	1520 (25.6)	347 (79.6)	
**Smoking**			
N	5867 (94.7)	371 (80.3)	< 0.0001
Y	288 (5.3)	77 (19.7)	
Height (cm)	158.07 ± 0.12	157.64 ± 0.43	0.331
Weight (kg)	58.33 ± 0.15	58.83 ± 0.67	0.470
BMI (kg/m^2^)	23.36 ± 0.06	23.67 ± 0.25	0.227
Fasting glucose (mg/dL)	97.43 ± 0.35	99.89 ± 1.15	0.034
SBP (mmHg)	115.20 ± 0.32	116.82 ± 0.88	0.071
DBP (mmHg)	73.40 ± 0.17	73.78 ± 0.49	0.443
HbA1c (%)	5.61 ± 0.01	5.68 ± 0.05	0.125
TC (mg/dL)	193.73 ± 0.58	192.68 ± 2.24	0.649
HDL-C (mg/dL)	54.97 ± 0.23	54.28 ± 0.8	0.400
TG (mg/dL)	114.29 ± 1.38	126.75 ± 4.82	0.011
LDL-C (mg/dL)	118.71 ± 1.58	114.01 ± 4.6	0.330
Hb (g/dL)	13.1 ± 0.02	13.09 ± 0.07	0.912
TC/HDL-C	3.69 ± 0.02	3.75 ± 0.07	0.358
LDL-C/HDL-C	2.77 ± 0.04	2.69 ± 0.11	0.511
TG/HDL-C	2.37 ± 0.04	2.70 ± 0.13	0.016

‘Y’ represents presence of perceived stress, and ‘N’ represents absence of stress.

‘Y’ represents current smokers and ‘N’ represents non-smokers.

‘Y’ represents current drinkers and ‘N’ represents non-drinkers.

Values are presented as number (%) or the mean ± standard deviation.

*p* value was determined through the complex sample Rao–Scott adjusted chi-square test and complex sample generalized linear model T test.

*PHQ-9* Patient Health Questionnaire-9, *BMI* body-mass index, *SBP* systolic blood pressure, *DBP* diastolic blood pressure, *HbA1c* hemoglobin A1c, *TC* total cholesterol, *HDL-C* high density lipoprotein cholesterol, *TG* triglyceride, *LDL-C* low density lipoprotein cholesterol, *Hb* hemoglobin.

**Table 3:** Relationship between lipid ratio and depression.

Variable	Men PHQ-9 ≧ 10		Women PHQ-9 ≧ 10	
	OR	*p*	OR	*p*
**Household income**				
Low	2.732 (1.512–4.936)	0.001	2.715 (1.787–4.126)	< 0.0001
Middle-low	1.115 (0.602–2.064)	0.729	2.016 (1.303–3.12)	0.002
Middle-high	0.635 (0.284–1.419)	0.267	1.863 (1.171–2.962)	0.009
High	1		1	
**Education level**				
Elementary	2.457 (1.236–4.881)	0.010	2.458 (1.603–3.769)	< 0.0001
Middle	1.007 (0.468–2.167)	0.986	1.709 (1.029–2.84)	0.039
High	0.857 (0.526–1.395)	0.534	1.034 (0.745–1.435)	0.842
College	1		1	
Age	0.9996 (0.983–1.017)	0.961	1.001 (0.988–1.014)	0.913
Stress perception (YES)	10.41 (6.88–15.751)	< 0.0001	12.034 (8.615–16.808)	< 0.0001
Smoking (YES)	1.405 (0.961–2.052)	0.079	3.219 (2.171–4.771)	< 0.0001
Fasting glucose (mg/dL)	0.999 (0.998–1.001)	0.431	1.001 (0.997–1.004)	0.752
TG/HDL-C	1.041 (1.004–1.079)	0.028	0.995 (0.902–1.097)	0.915

Adjusted for age, sex, education, alcohol, smoking.

The ORs and 95% CI are determined through the complex sample logistic regression test.

*PHQ-9* Patient Health Questionnaire-9, *HDL-C* high density lipoprotein cholesterol, *TG* triglyceride.

### Ethics approval

This study followed the ethical standards laid out in the Declaration of Helsinki and was approved by Wonkwang University Hospital’s clinical trial screening committee (IRB) (approval number: 2021-09-001).

### Consent to participate

The ethics committee waived the requirement for consent. We only reviewed the patient records for this study, and we guaranteed that we would not use the information for anything other than for research purposes. Additionally, we reviewed the records that provided only the identification numbers of participants instead of their names.

### Consent for publication

Not applicable. This is because all patient information was investigated anonymously, and the manuscript did not reveal the patients’ personal clinical information or their images.

## Results

### Participant characteristics

A total of 11,648 participants were included in this study. Of these, 5043 were men, and 6605 were women. Among the male participants, 4858 (96.3%) had a PHQ-9 score < 10, whereas 185 (3.7%) had a PHQ-9 score ≧ 10. Among the female participants, 6,156 (93.2%) had a PHQ-9 score < 10, whereas 449 (6.8%) had a PHQ-9 score ≧ 10 (Fig. 1). There were significant differences in income, stress, smoking history, FBG, TG, and TG/HDL-C levels between men with and without depression (Table 1). There were also significant differences in income, education, stress, smoking history, FBG, TG, and TG/HDL-C levels between women with and without depression (Table 2).

### Relationship between lipid ratio and depression

Factors that affect the probability of depression and the relationship between the lipid ratio and depression were analyzed after adjusting for variables. Among men, the probability of depression was 10.41 times higher in those with perceived stress than in those without perceived stress. An increase in TG/HDL-C by 1 was associated with a 1.041-fold higher probability of depression. Among women, the probability of depression was 12.034 times higher in those with perceived stress than in those without perceived stress, and the probability of depression in current smokers was 3.219 times higher than the probability of depression in non-smokers. Among men, TG/HDL-C was correlated with the probability of depression.

## Discussion

In this study, we examined the relationship between depression and three lipid ratios, namely TC/HDL-C, LDL-C/HDL-C, and TG/HDL-C. The results showed that there is an association between TG/HDL-C and depression in men. However, TC/HDL-C and LDL-C/HDL-C were not associated with depression in both men and women.

Previous studies have mainly focused on the relationship between the abovementioned lipid ratios and cardiovascular disease and insulin resistance^13–16^. However, no previous study has been conducted to analyze the association between these ratios and depression. Some studies have demonstrated that the TC/HDL-C ratio is an important marker of cardiovascular risk and is mainly associated with insulin resistance^13,14,16,17^. As shown by the Framingham risk score, the risk of coronary artery disease increases with increase in the TG/HDL-C ratio^12^. In addition, a high TG/HDL-C ratio predicts the presence of small and dense LDL particles, and is useful for diagnosing the onset of insulin resistance and metabolic syndrome^18^.

Previous studies have shown that low TC and LDL-C levels are correlated with the onset of depression^15,16,19,20^. There are some plausible explanations for this relationship. First, depression reduces a patient’s appetite, leading to a low serum TC level^19^. Second, cytokine activation, which interferes with cholesterol synthesis, occurs in depression^21^. Third, low cholesterol level can reduce the availability of serotonin, making the patient more susceptible to depression^16^.

A study conducted on 8390 people using data from the National Health Survey of the United States demonstrated an association between depression and cholesterol level. Depression was diagnosed using the PHQ-9, and LDL-C levels were divided into three groups: < 169 mg/dL, 169–221 mg/dL, and ≥ 222 mg/dL. The results showed that compared to the moderate group (169–221 mg/dL), the OR for depression was 5.13 (1.74–15.09) in the low group (< 169 mg/dL) and 2.28 (1.07–4.86) in the high group (222 mg/dL or higher), which showed a U-shaped relationship. However, LDL-C and HDL-C were not associated with moderate depression^15^. Although the LDL-C level is known to be associated with depression, our study identified a relationship between cholesterol ratio and depression, revealing the importance of evaluating lipid ratio.

Low cholesterol levels are associated with mental health indicators. The association between low cholesterol level and depression has been consistently proven in laboratory studies. In addition, less favorable or depressive behaviors were observed in studies of animals with low cholesterol levels^22,23^. In a previous report, patients who used cholesterol-lowering drugs long-term showed signs of depression^20^. These findings can be explained by the significant correlation between plasma serotonin and low cholesterol concentration, which has been previously reported^2,24^.

Similar to the present study, some previous studies showed that depression is associated with high TG and low HDL-C levels^25,26^. A recent meta-analysis performed to investigate whether lipid parameters differed between healthy individuals and patients with first-episode major depressive disorder revealed that elevated TG and decreased HDL-C levels are associated with first-episode major depressive disorder^25^. Although the TG and HDL-C levels are known to be associated with depression, our study identified a relationship between TG/HDL-C ratio and depression, revealing that evaluation of lipid ratio is crucial.

The serum lipid levels of people with depression and suicidal ideation have been analyzed in a previous study using KNHANES data, the results of which showed a significant association of high HDL-C and TG levels with depression^26^. However, unlike the present study, it was conducted using KNHANES data from 2014, and depression was diagnosed as a PHQ-9 score ≥ 5.

The potential mediators of depression in lipids or lipoproteins and their association with the heterogeneity of symptoms have been investigated in a previous study^27^. The results indicated that melancholic features are independently associated with low HDL-C level, whereas atypical depression is independently associated with high TC and LDL-C levels^27^.

The results of the present study are consistent with the findings of previous studies that suggest that depression is associated with metabolic syndrome. Research on the relationship between metabolic syndrome and depression has been actively conducted in recent years^6,28,29^. Several possible mechanisms may be behind this correlation between metabolic syndrome and depression. Individuals with depression are more likely to engage in unhealthy behaviors, such as smoking, drinking alcohol, unhealthy diet and lifestyle, and non-compliance with medical treatment, than those who are not^29^. In addition, depression causes dysregulation of the hypothalamic–pituitary–adrenal axis, which may explain its association with metabolic syndrome^30^. There is a study that analyzed whether CRP and TG were related to suicide attempts in patients with major depressive disorder. The results of the study showed that CRP was significantly higher in suicide attempters than in depressed patients who did not attempt suicide and in healthy controls. TG levels were significantly higher in depressed patients compared to healthy controls^31^.

The presence of depression was analyzed in this study using PHQ-9 scores. The usefulness of the PHQ-9 score as a diagnostic criterion for depression has already been verified in Korea and in other countries^7–9^. However, the score used as a diagnostic criterion varies from study to study. In the present study, a PHQ-9 score ≧ 10 was diagnosed as depression. In the study by Kroenke et al.^19^, a cutoff of 9 points had a high sensitivity of 95% and a specificity of 84%. However, 10 points, which is simple and easy to remember or apply in actual clinical settings, has been suggested as the optimal cutoff point^32^.

A review journal reports that the association between depression and lipid profile leads to several conclusions^33^. That is, some studies reported that the lower the cholesterol level, the more adversely affected depression, and other studies reported no relationship. Although our results cannot hypothesize an association between lipid profile and depression, it can be hypothesized that at least TG/HDL-C is associated with depression mediated by weight gain or poor lifestyle.

The present study is meaningful because it is the first to analyze the association between lipid ratios and depression in a large population. The results of this study indicate that TC/HDL-C and LDL-C/HDL-C are not associated with depression. However, this finding may have been influenced by the sample size, design, and participants of this study. Therefore, future large-scale prospective clinical studies are needed to verify this conclusion.

This study has some limitations. First, we used data from a study in which depression and lipid levels were measured only once. Depression and cholesterol levels are likely to fluctuate over time; thus, failure to account for these fluctuations may have clouded the observed association. Second, the data used in the study are from large-scale government surveys, and there were several limitations in statistical analysis. Degree and characteristics of depression, presence of suicidal ideation/attempts were not investigated. Additionally, administration of psychotropic medications or cognitive therapy were not investigated. Third, as this was a cross-sectional study, it was difficult to ascertain the relationship between low cholesterol level and depression.

## Conclusions

This study is the first to examine the association between depression and three lipid ratios, namely TC/HDL-C, LDL-C/HDL-C, and TG/HDL-C. The results showed an association between TG/HDL-C and depression in men. However, TC/HDL-C and LDL-C/HDL-C were not associated with depression in both men and women. Depression is not just a psychiatric disease, but a disease that needs to be approached in terms of metabolic abnormalities as well. In this study, we measured the lipid ratio in patients with depression and brought attention to mental health in individuals with abnormal lipid ratio. Future studies reflecting the limitations of our study may provide a clinical basis for lipid metabolism in depression.

## Data Availability

Publicly available datasets were analyzed in this study, which could be used after obtaining approval from the relevant authorities. The data can be found at https://knhanes.kdca.go.kr/knhanes/sub03/sub03_02_05.do.
